# A Rare Phenomenon of Pulseless Body Movements Induced During Prolonged Cardiopulmonary Resuscitation

**DOI:** 10.7759/cureus.40465

**Published:** 2023-06-15

**Authors:** Waseem M Ilyas, Charuta Gadkari, Akhilesh Singh, Gajanan Chavan

**Affiliations:** 1 Emergency Medicine, Jawaharlal Nehru Medical College, Datta Meghe Institute of Higher Education and Research, Wardha, IND; 2 Emergency medicine, Jawaharlal Nehru Medical College, Datta Meghe Institute of Higher Education and Research, Wardha, IND

**Keywords:** return of spontaneous circulation (rosc), cpric, resuscitation, pulseless consciousness, cardiopulmonary resuscitation-induced consciousness

## Abstract

Patients receiving cardiopulmonary resuscitation (CPR) may rarely experience cardiopulmonary resuscitation-induced consciousness (CPRIC), manifesting as body movements, eye-opening, or even awareness. We present a case report of a 55-year-old male patient who experienced CPRIC but did not survive despite resuscitative measures. The patient suffered a sudden cardiac arrest and received early initiation of CPR. However, CPRIC posed a treatment dilemma for our resuscitation team as the patient displayed body movements, requiring careful management to avoid interruptions in CPR. The challenge of differentiating CPRIC from the return of spontaneous circulation (ROSC) highlights the need for further research and evidence-based guidelines. Effective management strategies for CPRIC are necessary to guide resuscitation teams in making informed decisions. Understanding and addressing CPRIC can improve the quality of CPR and post-resuscitation care, supporting the well-being of both patients and healthcare providers. Further investigation is essential to developing comprehensive approaches to managing CPRIC and improving patient outcomes.

## Introduction

Cardiopulmonary resuscitation-induced consciousness (CPRIC) is a fascinating and rare phenomenon that has garnered attention in resuscitation medicine. It is described as consciousness during cardiopulmonary resuscitation (CPR), with any degree of activity ranging from just opening eyes to even actively grabbing and conversing with medical personnel, despite the absence of a return of spontaneous circulation [1-3]. In this case report, we present the dilemma faced by our resuscitation team when encountering CPRIC in a 55-year-old male patient who ultimately did not survive.

CPRIC poses a treatment challenge as it can be mistaken for a return of spontaneous circulation (ROSC), leading to inappropriate rhythm and pulse checks and potentially interrupting CPR unnecessarily [4]. In our patient, the resuscitation team struggled to differentiate between CPRIC and ROSC, which resulted in difficulties managing the patient’s conscious movements without compromising the quality of CPR. Guidelines and medications, such as ketamine, midazolam, propofol, etomidate, and fentanyl, have been proposed to manage patient agitation during CPRIC. However, the need for proper research leaves healthcare providers with limited evidence-based approaches. Effective management strategies for CPRIC must be explored and validated through further research to guide resuscitation teams in making appropriate treatment decisions [4].

## Case presentation

This case report concerns a 55-year-old male who presented to our Emergency Department (ED) complaining of severe active and persistent left-sided chest pain for six hours, not subsiding with analgesics. He was conscious and alert but irritable. He had a radial pulse rate of 74 per minute, an oxygen saturation of 95% in room air, and a respiratory rate of about 28 per minute. While his blood pressure was being checked, he suddenly became unresponsive with an absent carotid pulse suggestive of cardiopulmonary arrest, which occurred within just a few minutes of his arrival. Cardiopulmonary resuscitation was started per the latest 2020 advanced cardiovascular life support (ACLS) guidelines. The rhythm was persistently pulseless electrical activity (PEA) with a wide complex. Bag and mask ventilation, chest compressions, and adrenaline were administered in accordance with ACLS recommendations. The bedside glucose level was 180 mg/dl. The patient was intubated after about 20 minutes and connected to a ventilator.

After 40 minutes of resuscitation, when it seemed like all probability of resuscitation would fail, the patient started moving with the flexing and extending of both hands and legs with the opening of the eyes, with the monitor electrocardiogram (ECG) rhythm still showing a wide complex PEA (Video 1). At this point, resuscitation measures were stopped; however, the carotid pulse could not be palpated even by multiple resuscitation providers on the team, there were no appreciable breathing movements and blood pressure was not recordable. This situation was a dilemma in treatment, as no one on the team had encountered such an event. It was decided to continue chest compressions and adrenaline as per ACLS guidelines, with a few providers restraining him with their hands so that the blood supply to the brain was not compromised. After a further five minutes of resuscitation, again on examination, the carotid pulse could not be elicited, there was non-recordable blood pressure, and the patient was still exhibiting such movement. At this point, resuscitation measures were again stopped, and a 12-lead ECG was taken, which showed ST elevation in the anterior leads.

**Video 1:** Pulseless consciousness occurred after prolonged CPR

It was then decided to sedate and paralyze the patient with 3mg of midazolam and 4mg of vecuronium, respectively, following which the patient’s movements stopped, and resuscitation was continued. After a further 10 minutes of CPR, during which the patient had no pulse and no body movements, the ECG rhythm became asystole, and it remained as such for another 30 minutes of CPR, after which the patient was declared dead.

## Discussion

The presented case highlights a significant treatment dilemma encountered by the resuscitation team in managing motor movements that occurred in a patient in cardiac arrest undergoing cardiopulmonary resuscitation. Our patient exhibited eye opening and movement of limbs in the absence of ROSC. In searching the medical literature, it was found that these movements fit the description of CPRIC. CPRIC includes any level of consciousness with any degree of activity ranging from just opening eyes to even actively grabbing and conversing with medical personnel, despite the absence of a return of spontaneous circulation [1-6]. Whether the body movements exhibited by the patient could be included under “consciousness” in “cardiopulmonary resuscitation-induced consciousness” is debatable. However, the term “consciousness” itself is ambiguous. It includes even patients with lower scores on the Glasgow Coma Scale (GCS), having impaired levels of “consciousness,” and exhibiting decerebrate posturing, which is an involuntary extensor movement of the limbs [7-10].

Our resuscitation team faced challenges differentiating between CPRIC and ROSC, leading to confusion and perhaps inappropriate interventions. The patient’s motor movements during CPR posed a specific treatment dilemma, as balancing them with uninterrupted CPR and avoiding complications and obstacles in achieving ROSC was essential [1,2]. The causes of CPRIC are still unidentified. Different cerebral ischemia thresholds among patients and other physiological parameters, including autoregulation and comorbidities, have been put forth as potential causes [4]. According to case reports, this condition is more likely to occur in young male patients who had CPR resumed on them right away after a cardiac arrest and in patients whose initial heart rhythm was ventricular fibrillation or pulseless ventricular tachycardia [11]. Our patient’s clinical scenario was somewhat similar to a case report by Abboud and Varanasi, where the ECG was suggestive of anterior wall ST-elevation myocardial infarction (STEMI), similar to our patient’s ECG after CPRIC, which also showed changes suggestive of anterior wall STEMI [4].

Careful management of CPRIC is necessary to maintain efficient resuscitation while attending to the patient’s conscious condition. To reduce patient agitation during CPRIC, drugs such as ketamine, midazolam, propofol, etomidate, and fentanyl have been suggested [4]. However, the lack of controlled trials and evidence-based protocols for managing CPRIC contributes to the treatment dilemma faced by healthcare providers. Further study is required to determine whether these people should be physically restrained or sedated [2]. Further research, a review of reported cases, sedation and medication guidelines, and novel interventions are necessary to guide healthcare providers in making informed treatment decisions and improving outcomes for patients experiencing CPRIC.

## Conclusions

CPRIC poses unique challenges in resuscitation efforts, including the potential for confusion with ROSC. Further research is needed to develop effective management strategies and provide guidance for healthcare providers encountering CPRIC. Recognition and understanding of CPRIC can enhance resuscitation quality and improve post-resuscitation care for patients experiencing this rare phenomenon.
